# Ultrasound-Assisted Synthesis of Novel 4-(2-Phenyl-1,2,3-Triazol-4-yl)-3,4-Dihydropyrimidin-2(1*H*)-(Thio)ones Catalyzed by Sm(ClO_4_)_3_

**DOI:** 10.3390/molecules15042087

**Published:** 2010-03-24

**Authors:** Chen-Jiang Liu, Ji-De Wang

**Affiliations:** 1 School of Science, Xi’an Jiaotong University, 710049 Xi’an, China; E-Mail: pxylcj@126.com; 2 School of Chemistry and Chemical Engineering, Xinjiang University, Key Laboratory of Oil and Gas Fine Chemicals, Ministry of Education, 830046 Urumqi, China

**Keywords:** Biginelli reaction, dihydropyrimidinone, triazole, Sm(ClO_4_)_3_, ultrasound

## Abstract

An efficient synthesis of novel 4-(2-phenyl-1,2,3-triazol-4-yl)-3,4-dihydro-pyrimidin-2(1*H*)-(thio)ones from 1,3-dicarbonyl compounds, 2-phenyl-1,2,3-triazole-4-carbaldehyde and urea or thiourea under ultrasound irradiation and using samarium perchlorate as catalyst is described. Compared with conventional methods, the main advantages of the present methodology are milder conditions, shorter reaction times and higher yields.

## Introduction 

3,4-Dihydropyrimidin-2(1*H*)-ones and their derivatives have attracted increasing interest owing to their therapeutic and pharmaceutical properties, such as antiviral, antibacterial, anti-inflammatory and antitumour activities [1,2,3]. Recently, functionalized dihydropyrimidinones have been successfully used as antihypertensive agents, calcium channel blockers, adrenergic and neuropeptide Y (NPY) antagonists [4,5]. In addition, some alkaloids containing the dihydropyrimidine core unit which also exhibit interesting biological properties have been isolated from marine sources. Most notably, among these are the batzelladine alkaloids, which were found to be potent HIV gp-120-CD4 inhibitors [6,7]. 

The original protocol for the synthesis of dihydropyrimidinones, reported by Biginelli in 1893, involves a one-pot reaction of benzaldehyde, ethyl acetoacetate and urea in ethanol under strongly acidic conditions [8]. However, this method suffers from drawbacks such as low yields (20–40%) of the desired products, particularly in case of substituted aldehydes, and loss of acid sensitive functional groups during the reaction. This has led to multi-step synthetic strategies that produce somewhat better yields, but which lack the simplicity of the original one-pot Biginelli protocol [9]. The search for more suitable preparation of dihydropyrimidinones continues today.

Recently, many synthetic methods for preparing these compounds have been developed to improve and modify this reaction by using Lewis acid catalysts as well as protic acids including FeCl_3_·6H_2_O, NiCl_2_·6H_2_O [10], lanthanide triflate [11], H_3_BO_3_ [12], VCl_3_ [13], Sr(OTf)_2_ [14], PPh_3_ [15], Indium(III) halides [16], LiBr [17], Silicasulfuric acid [18], Mn(OAc)_3_·2H_2_O [19], Y(NO_3_)_3_·6H_2_O [20], In(OTf)_3_ [21], TaBr_5_ [22], Ce(NO_3_)_3_·6H_2_O [23], silica chloride [24], HCOOH [25], SrCl_2_·6H_2_O-HCl[26], Yb(OTf)_3_ [27], Bi(NO_3_)_3_·5H_2_O [28], tungstate sulfuric acid [29], HClO_4_-SiO_2_ [30] and so on. In addition, ionic liquids [31], microwave irradiation [32] and ultrasound irradiation [33] were also utilized as the catalytic condition. However, in spite of their potential utility, many of these methods involve expensive reagents, strong acidic conditions and long reaction times.

As environmental consciousness has increased in chemical research and industry, the challenge for a sustainable environment calls for clean procedures. Ultrasound has gradually been introduced in organic synthesis as a green synthetic approach over the last three decades. Compared with traditional methods, this technique is more convenient and easily controlled. A large number of organic reactions can be carried out under milder conditions, in shorter reaction times and providing higher yields under ultrasound irradiation [34].

**Scheme 1:**
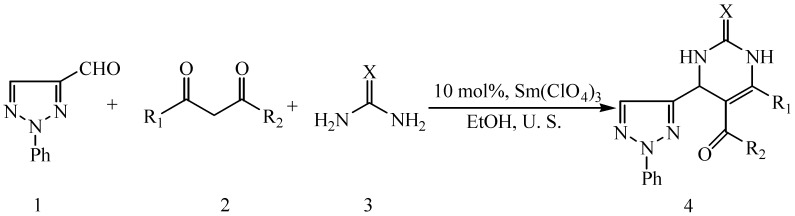
The synthesis of 4-(2-phenyl-1,2,3-triazol-4-yl)-3,4-dihydropyrimdin-2(1*H*)-(thio)ones catalyzed by Sm(ClO_4_)_3_.

Due to their unique biological properties, 1,2,3-triazole derivatives have attracted much attention [35]. In continuation of our interest in Lewis acid applications for dihydropyrimidinones and ultrasound-assisted synthesis [36], herein we report the preparation of 4-(2-phenyl-1,2,3-triazol-4-yl)-3,4-dihydropyrimidin-2(1*H*)-(thio)ones from 1,3-dicarbonyl compounds, 2-phenyl-1,2,3-triazole-4-carbaldehyde and urea or thiourea in the presence of Sm(ClO_4_)_3_ under ultrasound irradiation (Scheme 1). In this work, an efficient method for the synthesis of target compounds is described and none of them have been reported yet in the literature.

## Results and Discussion

To begin this study, different catalysts were tested in the condensation reactions of ethyl acetoacetate (2 mmol), 2-phenyl-1,2,3-triazole-4-carbaldehyde (2 mmol) and urea (3 mmol) in refluxing ethanol, affording 5-ethoxycarbonyl-6-methyl-4-(2-phenyl-1,2,3-triazol-4-yl)-3,4-dihydro-pyrimidin-2(1*H*)-one (**4a**) in various yields (Table 1). These results show that samarium perchlorate was the most efficient of the four catalysts studied. In order to identify the best molar ratio of reagents, we next did the experiment with different ratios of 1,3-dicarbonyl compounds, 2-phenyl-1,2,3-triazole-4-carbaldehyde, urea or thiourea and Sm(ClO_4_)_3_ in EtOH refluxing at 75–80 °C under ultrasonic irradiation. We found that a 1:1:1.5:0.1 molar ratio of reactants gave the best results.

**Scheme 2:**
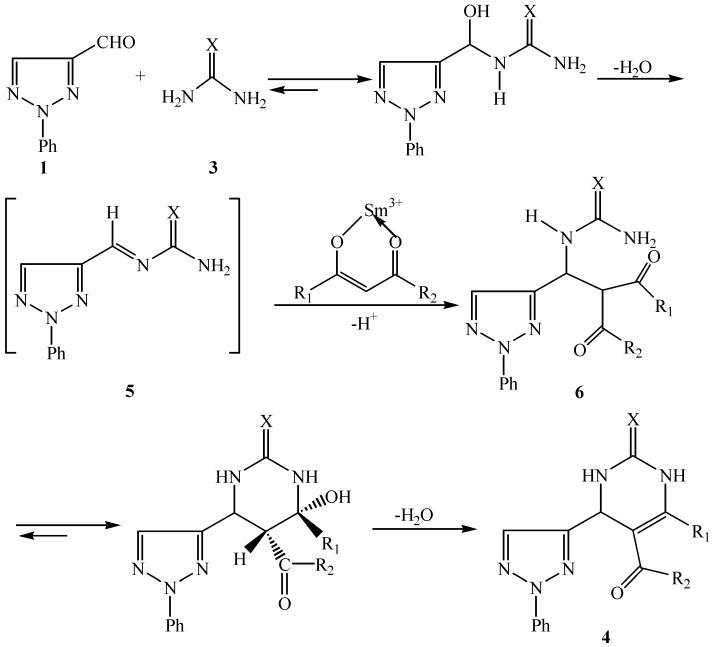
Mechanism of Biginelli reaction catalyzed by Sm(ClO_4_)_3_.

Using these optimized reaction conditions, the reaction of various 1,3-dicarbonyl compounds, 2-phenyl-1,2,3-triazole-4-carbaldehyde and urea or thiourea using Sm(ClO_4_)_3_ as catalyst under both ultrasound irradiation and conventional conditions were investigated. The results are summarized in Table 2. It was found that all the reactions proceeded smoothly to give the corresponding 4-(2-phenyl-1,2,3-triazol-4-yl)-3,4-dihydropyrimidin-2(1*H*)-(thio)ones in good yields. Compared to the conventional heating method, the achieved yields under ultrasound irradiation increase six or eight percent and only one-third of the reaction time need. According to the mechanism suggested by Kappe [37], a proposed reaction mechanism of Biginelli condensation *via* acyl imine intermediate **5** is presented in Scheme 2, this intermediate is formed by the reaction of the 2-phenyl-1,2,3-triazole-4-carbaldehyde **1** and urea or thiourea **3** and then stabilized by Sm(ClO_4_)_3_ through a coordinate bond owing to its empty orbital. Subsequent addition of the iminium ion to ethyl acetoactate in the presence of Sm(ClO_4_)_3_ as catalyst produces an open chain ureide **6** which subsequently undergo cyclization and dehydration to afford the corresponding dihydropyrimidinone **4**. 

## Experimental

### General 

All compounds were characterized by IR, ^1^H-NMR spectra and elemental analysis. The IR spectra were obtained as potassium bromide pellets with a FTS-40 spectrometer (BIO-RAD, USA). The ^1^H-NMR spectra were obtained on a Varian Inova-400 spectrometer using CDCl_3_ or DMSO-d_6_ as solvent (details given below) and TMS as an internal standard; chemical shifts are given in ppm. Elemental analyses (C, H, N) were performed on a Perkin-Elmer Analyzer 2400. Melting points were determined using a Büchi B-540 instrument. Sonication was performed in a Kunshan ultrasonic cleaner (KQ5200B, Kunshan Ultrasonic Instrument Co. Ltd.) with a frequency of 40 KHz and a nominal power of 200W. The reaction flask was located in the maximum energy area of the cleaner, where the surface of reactants is slightly lower than the level of the water. All melting points are uncorrected. 2-Phenyl-1,2,3-triazole-4-carbaldehyde was synthesized according to previous literature [38]. 

*General procedure for the synthesis of 4-(2-phenyl-1,2,3-triazol-4-yl)-3,4-dihydropyrimidin-2(1H)-(thio)ones* **4a-4k**

2-Phenyl-1,2,3-triazole-4-carbaldehyde (1 mmol), 1,3-dicarbonyl compound (1 mmol), urea or thiourea (1.5 mmol), Sm(ClO_4_)_3_ (10 mol %) and absolute EtOH (10 mL) were placed in a Pyrex flask (50 mL). The mixture was irradiated in the water bath of the ultrasonic cleaner at 75–80 °C for a specified period. Sonication was continued until the starting material had disappeared as indicated by TLC using ethyl acetate/petroleum ether (1:3) as eluent. After completion of the reaction, the mixture was cooled to room temperature and filtered. The collected solid was washed with a little amount of ethanol, and then dried. The products were pure enough without further purification. The results are summarized in Table 2. Data of the compounds are shown below:

*5-Ethoxycarbonyl-6-methyl-4-(2-phenyl-1,2,3-triazol-4-yl)-3,4-dihydropyrimidin-2(1H)-one* (**4a**): White solid. IR: ν 3296, 3179, 3079, 2984, 1679, 1643, 1600, 1452, 1370, 1144, 966, 761, 689 cm^-1^; ^1^H-NMR (CDCl_3_) δ: 10.32 (bs, 1H, NH), 9.55 (bs, 1H, NH), 7.63 (s, 1H, Tr-H), 7.32-8.02 (m, 5H, Ph), 5.72 (s, 1H, CH), 4.18 (q, *J =* 7.2 Hz, 2H, OCH_2_), 2.38 (s, 3H, CH_3_), 1.25 (t, *J =* 7.2 Hz, 3H, CH_3_*CH_2_O); Anal. calcd. for C_16_H_17_N_5_O_3_: C, 58.71; H, 5.23; N, 21.39. Found: C, 58.83; H, 5.20; N, 21.28. 

*6-Methyl-5-methoxycarbonyl-4-(2-phenyl-1,2,3-triazol-4-yl)-3,4-dihydropyrimidin-2(1H)-one* (**4b**): White solid. IR: ν 3307, 3182, 3090, 2972, 1667, 1638, 1611, 1449, 1389, 1128, 963, 770, 695 cm^-1^; ^1^H-NMR (CDCl_3_) δ: 10.31 (bs, 1H, NH), 9.29 (bs, 1H, NH), 7.86 (s, 1H, Tr-H), 7.39-7.95 (m, 5H, Ph), 5.44 (s, 1H, CH), 3.61 (s, 3H, OCH_3_), 2.26 (s, 3H, CH_3_); Anal. calcd. for C_15_H_15_N_5_O_3_: C, 57.50; H, 4.83; N, 22.35. Found: C, 57.65; H, 4.79; N, 22.46.

*5-Acetyl-6-methyl-4-(2-phenyl-1,2,3-triazol-4-yl)-3,4-dihydropyrimidin-2(1H)-one* (**4c**): White solid. IR: ν 3290, 3166, 3071,2983, 1683, 1645, 1552, 1335, 1145, 955, 753, 692 cm^-1^; ^1^H-NMR (DMSO-*d_6_*) δ: 10.49 (bs, 1H, NH), 9.57 (bs, 1H, NH), 7.84 (s, 1H, Tr-H), 7.40-7.97 (m, 5H, Ph), 5.54 (s, 1H, CH), 2.28 (s, 3H, CH_3_CO), 2.27 (s, 3H, CH_3_); Anal. calcd. for C_15_H_15_N_5_O_2_: C, 60.60; H, 5.09; N, 23.56. Found: C, 60.51; H, 5.15; N, 23.62.

*6-Methyl-5-benzoyl-4-(2-phenyl-1,2,3-triazol-4-yl)-3,4-dihydropyrimidin-2(1H)-one* (**4d**): White solid. IR: ν 3287, 3172, 3076, 2984, 1688, 1640, 1601, 1333, 1149, 973, 763,700 cm^-1^; ^1^H-NMR (DMSO-*d_6_*) δ: 10.45 (bs, 1H, NH), 9.73 (bs, 1H, NH), 7.95 (s, 1H, Tr-H), 7.42-7.98 (m, 10H, Ph), 5.58 (s, 1H, CH), 1.67 (s, 3H, CH_3_CO); Anal. calcd. for C_20_H_17_N_5_O_2_: C, 66.84; H, 4.77; N, 19.49. Found: C, 66.94; H, 4.83; N, 19.38.

*5-Ethoxycarbonyl-6-phenyl-4-(2-phenyl-1,2,3-triazol-4-yl)-3,4-dihydropyrimidin-2(1H)-one* (**4e**): White solid. IR: ν 3292, 3164, 3079, 2980, 1673, 1641, 1591, 1550, 1454, 1375, 1147, 958, 761, 701 cm^-1^; ^1^H-NMR (DMSO-*d_6_*) δ: 10.43 (bs, 1H, NH), 9.70 (bs, 1H, NH), 8.00 (s, 1H, Tr-H), 7.33-7.98 (m, 10H, Ph), 5.56 (s, 1H, CH), 3.79 (q, *J =* 7.2 Hz, 2H, OCH_2_), 0.77 (t, *J =* 7.2 Hz, 3H, CH_3_*CH_2_O); Anal. calcd. for C_21_H_19_N_5_O_3_: C, 64.77; H, 4.92; N, 17.98. Found: C, 64.69; H, 4.86; N, 18.10.

*5-Ethoxycarbonyl-6-methyl-4-(2-phenyl-1,2,3-triazol-4-yl)-3,4-dihydropyrimidin-2(1H)-thione* (**4f**): White solid. IR: ν 3298, 3174, 3081, 2980, 1680, 1650, 1601, 1457, 1373, 1200, 1140, 960, 756, 696 cm^-1^; ^1^H-NMR (DMSO-*d_6_*) δ: 10.39 (bs, 1H, NH), 9.61 (bs, 1H, NH), 7.88 (s, 1H, Tr-H), 7.54-7.94 (m, 5H, Ph), 5.47 (s, 1H, CH), 4.07 (q, *J =* 7.2 Hz, 2H, OCH_2_), 2.30 (s, 3H, CH_3_), 1.13 (t, *J =* 7.2 Hz, 3H, CH_3_*CH_2_O); Anal. calcd. for C_16_H_17_N_5_O_2_S: C, 55.96; H, 4.99; N, 20.39. Found: C, 55.85; H, 5.02; N, 20.30.

*6-Methyl-5-methoxycarbonyl-4-(2-phenyl-1,2,3-triazol-4-yl)-3,4-dihydropyrimidin-2(1H)-thione* (**4g**): White solid. IR: ν 3279, 3139, 3070, 2973, 1675, 1641, 1603, 1472, 1369, 1206, 1129, 951, 767, 687 cm^-1^; ^1^H-NMR (DMSO-*d_6_*) δ: 10.45 (bs, 1H, NH), 9.69 (bs, 1H, NH), 7.89 (s, 1H, Tr-H), 7.40-7.95 (m, 5H, Ph), 5.47 (s, 1H, CH), 3.62 (s, 3H, OCH_3_), 2.32 (s, 3H, CH_3_); Anal. calcd. for C_15_H_15_N_5_O_2_S: C, 54.70; H, 4.59; N, 21.26. Found: C, 54.58; H, 4.65; N, 21.33.

*5-(tert-Butoxycarbonyl)-6-methyl-4-(2-phenyl-1,2,3-triazol-4-yl)-3,4-dihydropyrimidin-2(1H)-thione* (**4h**): White solid. IR: ν 3283, 3149, 3076, 2969, 1683, 1643, 1606, 1461, 1362, 1201, 1133, 949, 770, 679 cm^-1^; ^1^H-NMR (DMSO-*d_6_*) δ: 10.40 (bs, 1H, NH), 9.66 (bs, 1H, NH), 7.89 (s, 1H, Tr-H), 7.41-7.96 (m, 5H, Ph), 5.43 (s, 1H, CH), 2.29 (s, 3H, CH_3_), 1.38 (s, 9H, OC(CH_3_)_3_); Anal. calcd. for C_18_H_21_N_5_O_2_S: C, 58.20; H, 5.70; N, 18.85. Found: C, 58.37; H, 5.63; N, 18.76.

*5-Acetyl-6-methyl-4-(2-phenyl-1,2,3-triazol-4-yl)-3,4-dihydropyrimidin-2(1H)-thione* (**4i**): White solid. IR: ν 3291, 3177, 3072, 2980, 1681, 1643, 1560, 1336, 1205, 1145, 959, 762, 670 cm^-1^; ^1^H-NMR (DMSO-*d_6_*) δ: 10.52 (bs, 1H, NH), 9.63 (bs, 1H, NH), 7.84 (s, 1H, Tr-H), 7.40-7.94 (m, 5H, Ph), 5.56 (s, 1H, CH), 2.33 (s, 3H, CH_3_CO), 2.28 (s, 3H, CH_3_); Anal. calcd. for C_15_H_15_N_5_OS: C, 57.49; H, 4.82; N, 22.35. Found: C, 57.39; H, 4.77; N, 22.24.

*6-Methyl-5-benzoyl-4-(2-phenyl-1,2,3-triazol-4-yl)-3,4-dihydropyrimidin-2(1H)-thione* (**4j**): White solid. IR: ν 3290, 3169, 3077, 2985, 1688, 1639, 1601, 1334, 1203, 1136, 965, 750, 671 cm^-1^; ^1^H-NMR (DMSO-*d_6_*) δ: 10.36 (bs, 1H, NH), 9.71 (bs, 1H, NH), 7.94 (s, 1H, Tr-H), 7.39-7.97 (m, 10H, Ph), 5.59 (s, 1H, CH), 1.72 (s, 3H, CH_3_); Anal. calcd. for C_20_H_17_N_5_OS: C, 63.98; H, 4.56; N, 18.65. Found: C, 64.08; H, 4.53; N, 18.76.

*5-Ethoxycarbonyl-6-phenyl-4-(2-phenyl-1,2,3-triazol-4-yl)-3,4-dihydropyrimidin-2(1H)-thione* (**4k**): White solid. IR: ν 3294, 3174, 3079, 2979, 1679, 1643, 1599, 1554, 1456, 1372, 1201, 1140, 964, 757, 696 cm^-1^; ^1^H-NMR (CDCl_3_) δ: 10.38 (bs, 1H, NH), 9.67 (bs, 1H, NH), 7.78 (s, 1H, Tr-H), 7.35-8.08 (m, 10H, Ph), 5.86 (s, 1H, CH), 3.57 (q, *J =* 7.2 Hz, 2H, OCH_2_), 0.91 (t, *J =* 7.2 Hz, 3H, CH_3_*CH_2_O); Anal. calcd. for C_21_H_19_N_5_O_2_S: C, 62.21; H, 4.72; N, 17.27. Found: C, 62.11; H, 4.79; N, 17.36.

## Conclusions

In conclusion, we have successfully demonstrated an efficient protocol for the synthesis of 4-(2-phenyl-1,2,3-triazol-4-yl)-3,4-dihydropyrimidin-2(1*H*)-(thio)ones by the Sm(ClO_4_)_3_-catalyzed reaction of 1,3-dicarbonyl compounds, 2-phenyl-1,2,3-triazole-4-carbaldehyde and urea or thiourea under ultrasound irradiation. The protocol offers several advantages such as mild reaction conditions, short reaction times, easy isolation and good yields. Hence, it is an important supplement to the existing methods for the synthesis of dihydroprimidiones and their corresponding thio-derivatives.

## Figures and Tables

**Table 1:** Effects of different catalysts on the formation of **4a** under conventional conditions. ^a^

No.	Catalyst	Catalyst (mol %)	Time (h)	Yield of 4a^b^ (%)
**1**	HCl	10	24	42
**2**	Zn(ClO_4_)_2_	10	12	51
**3**	Mg(ClO_4_)_2_	10	12	70
**4**	Sm(ClO_4_)_3_	10	9	88

^a^ Reaction conditions: 2-phenyl-1,2,3-triazole-4-carbaldehyde (2 mmol), ethyl acetoacetate (2 mmol), urea (3 mmol), EtOH (10 mL), 75-80 °C; ^b^ Isolated yield.

**Table 2:** Sm(ClO_4_)_3_-catalyzed one-pot three-component synthesis of 4-(2-phenyl-1,2,3-triazol-4-yl)-3,4-dihydropyrimidin-2(1*H*)-(thio)ones.

Entry	R_1_	R_2_	X	Time (h)^a^		Yield (%)^b^		Mp ( °C)
				A^c^	B^c^	A^c^	B^c^	
**4a**	CH_3_	OCH_2_CH_3_	O	9	3	88	95	176-177
**4b**	CH_3_	OCH_3_	O	9	3	71	78	215-218
**4c**	CH_3_	CH_3_	O	6	2	89	94	245-246
**4d**	CH_3_	Ph	O	6	2	78	85	244-246
**4e**	Ph	OCH_2_CH_3_	O	9	3	72	80	203-206
**4f**	CH_3_	OCH_2_CH_3_	S	9	3	70	77	203-205
**4g**	CH_3_	OCH_3_	S	9	3	69	76	219-222
**4h**	CH_3_	OC(CH_3_)_3_	S	9	3	86	89	200-203
**4i**	CH_3_	CH_3_	S	6	2	76	84	269-270
**4j**	CH_3_	Ph	S	6	2	90	95	259-261
**4k**	Ph	OCH_2_CH_3_	S	9	3	80	86	204-206

^a^ Reactions were continued until the TLC shown the starting materials disappeared; ^b^ Isolated yield; ^c^ Method A: Sm(ClO_4_)_3_ as catalyst in refluxing EtOH at 75–80 °C without ultrasound; Method B: Sm(ClO_4_)_3_ as catalyst in refluxing EtOH at 75–80 °C under ultrasound irradiation.
